# Leveraging Community Pharmacies to Address Social Needs: A Promising Practice to Improve Healthcare Quality

**DOI:** 10.3390/pharmacy12050139

**Published:** 2024-09-11

**Authors:** Tony Kuo, Noel C. Barragan, Steven Chen

**Affiliations:** 1 Department of Family Medicine, David Geffen School of Medicine at University of California, Los Angeles (UCLA), Los Angeles, CA 90095, USA; 2 Department of Epidemiology, UCLA Fielding School of Public Health, Los Angeles, CA 90095, USA; 3 Population Health Program, UCLA Clinical and Translational Science Institute, Los Angeles, CA 90095, USA; 4 Division of Chronic Disease and Injury Prevention, Los Angeles County Department of Public Health, Los Angeles, CA 90010, USA; nbarragan@ph.lacounty.gov; 5 Titus Family Department of Clinical Pharmacy, Alfred E. Mann School of Pharmacy and Pharmaceutical Sciences, University of Southern California, Los Angeles, CA 90089, USA; chens@usc.edu

**Keywords:** community pharmacies, pharmacists, social needs, workforce development, healthcare quality

## Abstract

Emerging research suggests that chronic conditions such as cardiovascular disease, diabetes, and asthma are often mediated by adverse social conditions that complicate their management. These conditions include circumstances such as lack of affordable housing, food insecurity, barriers to safe and reliable transportation, structural racism, and unequal access to healthcare or higher education. Although health systems cannot independently solve these problems, their infrastructure, funding resources, and well-trained workforce can be realigned to better address social needs created by them. For example, community pharmacies and the professionals they employ can be utilized and are well-positioned to deliver balanced, individualized clinical services, with a focus on the whole person. Because they have deep roots and presence in the community, especially in under-resourced neighborhoods, community pharmacies (independent and chain) represent local entities that community members recognize and trust. In this article, we provide case examples from California, United States, to illustrate and explore how community pharmacies can be leveraged to address patient social needs as part of their core responsibilities and overall strategy to improve healthcare quality.

## 1. Introduction

Emerging evidence suggests that chronic conditions such as cardiovascular disease, diabetes, and asthma are often mediated by adverse social conditions that complicate their management [1,2,3,4]. These conditions include circumstances such as lack of affordable housing, food insecurity, barriers to safe and reliable transportation, structural racism, and unequal access to healthcare or higher education [3,5,6]. Although health systems cannot independently solve these problems, they can realign their infrastructure, funding investments, and workforce to better address the social needs created by them [6,7,8]. There are a number of opportunities that health systems, particularly community pharmacies, can undertake to address social needs.

While feasible, realignment of care structure and resources is inherently challenging, as there are a number of existing barriers to initiating this action—e.g., physician shortages, low reimbursements for preventive and social services, lack of time during clinic visits to address patient social needs [6,8]. Increased use of team-based care is a potential solution and promising practice for mitigating some of these barriers. Team-based care is an approach for coordinating and delivering care that centers on patients’ needs, generally through collaborative communication and actions taken by a team of interdisciplinary healthcare providers. Expanding the role of pharmacists within care teams, for example, is particularly advantageous due to their expertise, high levels of established patient trust, and ubiquity. Increasingly, states have started adopting health policies that create structural frameworks and opportunities for shifting fragmented care to value-based models that leverage the clinical services of pharmacists. These models are grounded in multidisciplinary team care principles and address social needs as a key strategy for improving healthcare quality [5,9]. For example, as of 2014, legislation in California allows for pharmacists to engage in advanced practice pharmacist duties, including management of chronic conditions through team care models and promising practices such as comprehensive medication management (CMM) [10,11,12]. Subsequently, California Medicaid began allowing for billing of clinical services and for other support services delivered by pharmacists, pharmacy technicians, and community health workers (CHWs) [13]. These health policies represent critical developments that are presently being used to sustain and scale more holistic health interventions designed to support the ‘whole person’.

Experiences during the coronavirus disease 2019 (COVID-19) pandemic further brought awareness and attention to the community pharmacy infrastructure that was previously underrecognized in the United States (U.S.); the infrastructure could serve as a support for enhancing multidisciplinary team care. For instance, the engagement of community pharmacies (independent and chain) proved to be an efficient and reliable strategy for delivering vaccinations among priority populations (low-income, communities of color, those with disabilities) throughout the pandemic. Community members were familiar with and trusted these local pharmacies which they regularly visit. These pharmacies, in turn, were able to address vaccine hesitancy by helping patients disentangle mixed information about the vaccine’s effectiveness and safety [14,15]. Table 1 offers examples of opportunities where community pharmacies can play a role in addressing social needs and catalyzing change in health and social care delivery.

In this article, we provide case examples from California, United States, to illustrate and explore opportunities where community pharmacies can leverage their deep roots and presence in the community to expand patient-centered care—i.e., services and support that center around the whole person. We also explore how these pharmacies can strengthen community–clinical linkages to address patient social needs.

## 2. Physical Locations, a Ready Workforce, and Opportunities to Strengthen Community-Clinical Linkages

### 2.1. Physical Locations of Community Pharmacies Are Relatively Ubiquitous and Frequently Visited by Community Members

Community pharmacies are in a unique position to effectively address and manage social needs because they are relatively ubiquitous and frequently visited by community members. Pharmacies are located in every community, with 88.9% and 73.1% of the U.S. population living within 5 and 2 miles of a pharmacy, respectively [16]. In addition, patients visit their pharmacies, on average, 12 times more frequently than their primary care provider in a given year, providing abundant opportunities to offer health screenings, disease monitoring, and follow-up on treatment adherence [16,17,18]. Based on national statistics [18], Medicaid patients set foot in community pharmacies, on average, two to three times a month, while Medicare patients enter pharmacies at least once a month. Collectively, these geographic and clinical visit patterns suggest community pharmacies are well positioned to assess and address patients’ complex priorities, including social needs that can interfere with treatment success.

### 2.2. A Ready Workforce

Pharmacists employed by community pharmacies represent a highly trained workforce that can be leveraged to improve healthcare quality in the U.S. In California alone, there are more than 6000 retail pharmacies, nearly 50,000 pharmacists, and more than 65,000 pharmacy technicians spread out across both rural and urban communities (see Table 2 for a summary of these licensed professionals’ general and workplace characteristics). Because of their training and clinical experience, they can be readily prepared to manage patients with complex needs, and through multidisciplinary team care, connect patients to locally accessible social services and community resources when needed [11,12,14,15,17]. Recognizing this opportunity, the University of Southern California Alfred E. Mann School of Pharmacy and Pharmaceutical Sciences’ (USC Mann’s) California Right Meds Collaborative (CRMC) has been actively supporting innovative efforts to fully equip pharmacies and pharmacists to improve chronic disease management in diverse communities locally and across the state. CRMC is a consortium of health plans, pharmacies, community clinics, and academic and professional organizations that are dedicated to expanding California’s clinical pharmacist workforce. The Collaborative offers trainings and technical assistance to community pharmacies and pharmacists in support of their care delivery, continuous quality improvement (CQI) efforts, and business goals [19]. As an example, with CRMC’s support, Los Angeles County’s largest Medicaid health plan piloted and successfully established a value-based payment model to reimburse pharmacists who deliver CMM services (Box 1) [11,20,21,22]; these providers are paid for achieving blood pressure (BP) and other chronic disease targets among their priority populations. Since the model’s launch in 2019, the number of participating pharmacies has expanded to 18 sites; they have provided CMM services to >1100 patients, achieving BP control for 94% of those who completed at least five encounter visits [23].

Box 1 **Comprehensive medication management is a promising practice for addressing the complex needs of the whole person.**

- Comprehensive medication management (CMM) is a team-based care model that utilizes evidence-based strategies to manage medication selection, titration, monitoring, follow-up, and treatment adherence for patients with diagnosed chronic conditions, such as hypertension, diabetes, and asthma.
- This model of practice [11,23] represents a multistep longitudinal standard of care in which patients are:
  (a) Individually assessed to determine if their medications, including prescriptions, over-the-counter drugs, supplements, and alternative therapies, are appropriate, effective for the condition(s) they are treating, able to be taken as intended, and safe to use given identified comorbidities and drug-drug interaction(s).
  (b) Provided with an individualized care plan that achieves the intended goals of therapy, including interval medication titration.
  (c) Given appropriate follow-up to achieve actual patient outcomes.
- Because the model’s primary focus is on patient safety and addressing the complex needs of the whole person, CMM integrates protocols and/or procedures that are relevant and could be leveraged to assess and address social needs.
- CMM has been shown to make a significant impact on health outcomes. For example, in a 2012–2015 demonstration project funded by the Centers for Medicare and Medicaid Innovation, 87% of Los Angeles County participants achieved systolic and diastolic blood pressure readings of <140/90 mmHg within 3 months of starting the intervention. The program identified and resolved over 67,000 medication-related problems among 6000+ patients, including suboptimal medication selection, cost/formulary issues, lack of monitoring, and inadequate dosing [24].

**Table 2:** General characteristics of California pharmacies and their licensed professionals, 2023–2024.

Characteristic	Number
Pharmacy type ^a^	
Clinic pharmacy	2342
Correctional pharmacy	4
Hospital pharmacy	476
Government-owned pharmacy	144
Nonresident pharmacy (ships/mails/delivers to state residents)	599
Retail pharmacy (independent and chain)	6091
Pharmacy professional by license type ^a^	
Pharmacy technician	65,218
Intern pharmacist	4740
Pharmacist	49,906
Advanced practice pharmacist	1210
Estimated number of pharmacists employed in California in 2023–2024 ^b^	32,800
Industries that employ pharmacists ^b^	Number of employers in the state
Employment services	5110
Insurance carriers	855
Grocery and convenience retailer	15,069
General medical and surgical hospitals	1477
Physician offices	59,067
Outpatient care centers	10,342
No. of schools of pharmacy and training programs in California ^c^	14
California Practice Standards and Jurisprudence Examination pass rates among pharmacists trained by California Schools of Pharmacy ^c^	52.9%

^a^ Data source: California State Board of Pharmacy. License Type. https://www.pharmacy.ca.gov/about/license_total.shtml, accessed on 13 July 2024 [25]. ^b^ Data source: State of California Employment Development Department. Occupation Profile. Industries Employing Pharmacists. https://labormarketinfo.edd.ca.gov/cgi/databrowsing/occExplorerQSDetails.asp?soccode=291051, accessed on 13 July 2024 [26]. ^c^ Data source: California State Board of Pharmacy, California State Board of Pharmacy CPJE Statistics. 1 October 2023—31 March 2024. https://www.pharmacy.ca.gov/publications/1023_0324_stats.pdf, accessed on 13 July 2024 [27].

In addition to pharmacists, most community pharmacies also have pharmacy technicians and other support staff who can help patients with health and social services navigation. Many of them are culturally and linguistically aligned with the community they serve. In particular, pharmacy technicians frequently live in the same neighborhood and speak the same languages as their patients; they are trained to provide supportive services similar to those being offered by CHWs [28]. This workforce stands ready to assist patients with their chronic disease prevention and management needs, presenting ample and convenient opportunities to further advance chronic disease care. The potential involvement of pharmacy technicians in coordinating health and social services also aligns well with emerging policies. For example, state Medicaid programs like Medi-Cal in California are beginning to reimburse for these services—i.e., CHWs and nonphysician clinicians who function in a similar capacity (pharmacy technicians fall in this category) are eligible to bill for services delivered [13]. To leverage this unprecedented opportunity, USC Mann recently launched a professional development program to certify pharmacy technicians as CHWs in the state; the certification meets both state and national standards. Pharmacy technicians certified as CHWs can help pharmacy clients with navigating services and resources that are often necessary to maintain or improve treatment adherence. These services/resources may include food assistance, childcare, and legal aid for immigration problems.

### 2.3. Community Pharmacies as a Hub for Health-Related Activities in the Neighborhood

Community pharmacies hold tremendous promise beyond their familiar role as an entity that dispenses medications. They are increasingly becoming important points of care in neighborhoods across the U.S. [29]. Many of them already serve as a trusted hub for health-related activities in the neighborhood, where community members come for services, seek health advice, and receive counseling on disease prevention [29,30]. Because pharmacists, pharmacy technicians, and other support staff are highly trained and have the prerequisite knowledge, technical skills, and clinical experience to engage in this work, pharmacies can optimize and redirect their staff’s skills to address social needs, adding this practice to their repertoire. Similarly, their deep roots and presence in the community can be leveraged to build and tailor stronger community–clinical linkages for any given neighborhood, thereby connecting patients with social needs to appropriate social services and community resources. Capitalizing on community pharmacies’ strengths could yield cost savings from less emergency room visits and hospitalizations due to disease exacerbations triggered by adverse social factors.

## 3. The Emerging Problem of Pharmacy Deserts Also Highlights the Value of and Need for Community Pharmacies 

Another lens by which we can view the value of community pharmacies is the potential adverse impact their absence can have on vulnerable communities. Termed “pharmacy deserts”, this void in services—often the result of pharmacy closure—is particularly pronounced in rural areas and in areas with high densities of priority populations [31,32]. It can lead to a loss of or diminished access to diagnostic (e.g., testing for strep throat, flu, COVID-19), preventive (e.g., BP screening; counseling on alcohol misuse, depression, smoking cessation; vaccinations for influenza, respiratory syncytial virus, COVID-19, zoster, hepatis A and B), and emergency services (e.g., dispensing naloxone to reverse opioid overdose; contraceptives for preventing unplanned pregnancy). In a recent study of the 30 most populous U.S. cities, researchers found that, during 2007–2015, persistently fewer pharmacies were located in Black and Latino neighborhoods than in white or other diverse neighborhoods; these neighborhoods were also more likely to experience pharmacy closures [32]. Collectively, these two pharmacy trends have led to an increase in pharmacy deserts across the 30 cities in the U.S., from 8082 in 2007 to 9346 in 2015, affecting nearly 15 million people.

## 4. Implications for Policy and Practice

Mounting evidence suggests that a ‘whole person care’ approach may be required to improve healthcare quality in the U.S. and to effectively control chronic conditions such as cardiovascular disease, diabetes, and asthma [33,34,35]. Emerging evidence, including experiences from the COVID-19 pandemic, coalesces around better utilization and leveraging of existing pharmacy networks that are trusted by community members. These existing networks represent key opportunities for centering care around patients’ physical health, mental health, and social needs. Indeed, case examples like those in California offer practical lessons from the field, showing that community pharmacies and their use of multidisciplinary team care, like CMM, is a promising practice. Ultimately, unmet social needs, particularly for priority populations in underserved communities, are major contributors to poor chronic disease control and health inequities [35]. Solutions to this problem are urgently needed to coordinate patient-centered care that is comprehensive, compassionate, and convenient. To this point, community pharmacies represent a viable solution. Health systems, healthcare providers, and policymakers can and should capitalize on these emerging opportunities to integrate pharmacies into their community and healthcare planning, leveraging them whenever possible to achieve the quadruple aims of healthcare quality [34].

## 5. Conclusions

Current data and practice trends suggest that while community pharmacies are well-positioned to assess and address social needs and to coordinate complex care, many could benefit from more focused training, structural support, and funding to successfully implement these actions [36]. To make this endeavor a reality, intentional actions should be taken to: (i) strategically realign health-related resources; (ii) increase investments in CMM and other team care approaches; (iii) promote growth of value-centered collaboratives like CRMC so they can fully support workforce development through training and CQI support; and (iv) adopt value-based health policies that responsibly reimburse for services that are needed to assist patients with complex needs.

As community pharmacies expand their roles in patient care, many of these calls to action will require strategy and careful planning to harness political support for the movement and to find common ground with other health professions so that their presence is seen as complementary and not as competition. Efforts to implement a more user-friendly billing structure and/or to scale a value-based payment model tailored to the capabilities of these pharmacies will also be important for realizing these entities’ vast potential as trusted services providers and fixtures in the community.

## Figures and Tables

**Table 1:** Opportunities for community pharmacies to contribute or intervene on social needs across multiple sectors ^a^.

Sector	Social Needs Created by Adverse Social Conditions or Circumstances	Opportunities Where Community Pharmacies Can Contribute or Intervene
Child and Maternal Health Family planning and prenatal care. Health behaviors. Health insurance coverage.	Inadequate access to family planning services and prenatal care. High community prevalence of tobacco, alcohol, and drug use behaviors. Low enrollment in health insurance coverage.	Offer alternative sites where people can access family planning services and prenatal care (includes folic acid supplementation)—e.g., at pharmacies, non-primary care facilities. Expand public sector health insurance coverage (e.g., Medicaid) and increase outreach to enroll eligible groups (community pharmacies can serve as a physical hub for health insurance enrollment). Counsel clients (patients) on tobacco, alcohol, and drug use (pharmacists can provide these types of counseling). Trusted entities in the community like schools, faith-based organizations, pharmacies, and other community-based agencies regularly assess and address social needs, referring clients (patients) with these needs to appropriate social services and community resources.
Caregiving Caregiver support.	Poor awareness of or access to caregiver support resources.	Help raise public awareness about caregiver health and needs. Trusted entities in the community like schools, faith-based organizations, pharmacies, and other community-based agencies regularly serve as a resource or source of information for community members struggling with caregiving responsibilities.
Healthcare Healthcare access and quality. Complex care coordination, with a focus on the whole person and their competing priorities (e.g., social needs). Mental health services availability. Addiction treatment services availability.	Poor access to and use of affordable quality healthcare. Low enrollment in health insurance coverage. Complex care coordination/navigation services are not available to those who need them. Mental health services are not readily available. Addiction treatment services are not readily available.	Strengthen health policies and prioritize spending to ensure health, mental health, and addiction treatment services are available to priority populations. Expand public sector health insurance coverage (e.g., Medicaid) and increase outreach to enroll eligible groups (community pharmacies can serve as a physical hub for health insurance enrollment). Expand the scope of practice for emerging occupations and professions (pharmacists, pharmacy technicians, social workers, CHWs, etc.) that can take on a larger role in providing clinical and social services. Trusted entities in the community like schools, faith-based organizations, pharmacies, and other community-based agencies regularly assess and address social needs, referring clients (patients) with these needs to appropriate social services and community resources. Provide administration and monitoring of long-acting injectable antipsychotic medications (a role that pharmacies and pharmacists can play). Provide medication-assisted treatment for substance use (a role that pharmacies and pharmacists can play).
Housing Persons experiencing homelessness (PEH). Street medicine/psychiatric home teams.	Limited access to interventions like street medicine/psychiatric home teams for PEH.	Invest in and further expand programs like street medicine/psychiatric home teams, including leveraging community pharmacies to help address these unhoused populations’ individual health, mental health, and social needs, and reintegrate them into society.
Social Welfare Programs and Structure WIC, SNAP, SNAP-Ed, national school breakfast and lunch programs. Nutrition incentive programs. Food as Medicine Strategy interventions.	Food insecurity. Poor nutrition.	Expand access to WIC, SNAP, school breakfast and lunch programs (e.g., allow immigrant families and transitional youth to access SNAP program benefits). Support and expand Food as Medicine Strategy interventions, including medically tailored meals, produce prescriptions, and produce pharmacies. Trusted entities in the community like schools, faith-based organizations, pharmacies, and other community-based agencies regularly assess and address social needs, referring clients (patients) with these needs to appropriate social programs and community resources.

Priority populations: Those that are low-income, African-American, Latino, and/or Asian/Pacific Islander. K-12, kindergarten to grade twelve (school); SNAP, Supplemental Nutrition Assistance Program; SNAP-Ed, Supplemental Nutrition Assistance Program Education; WIC, Special Supplemental Nutrition Program for Women, Infants, and Children. ^a.^ Data and information for this table were drawn from viewpoints and studies in the scientific literature [3,4,8].

## Data Availability

Not applicable. In this article, the authors discuss publicly available data and information.
